# MYB59 is Linked to Natural Variation of Water‐Use Associated With Warmer Temperatures in *Arabidopsis thaliana*

**DOI:** 10.1002/pei3.70199

**Published:** 2026-08-09

**Authors:** John N. Ferguson, Oliver Brendel, Ulrike Bechtold

**Affiliations:** ^1^ School of Life Sciences University of Essex Colchester UK; ^2^ Université de Lorraine, AgroParisTech, INRAe, Silva Nancy France; ^3^ Department of Biosciences Durham University Durham UK

**Keywords:** adaptation, *Arabidopsis thaliana*, GWAS, *MYB59*, natural variation, plant water relations, water use

## Abstract

Water‐use contributes significantly to plant growth and productivity, yet the extent to which variation in water‐use is a function of adaptive differentiation is unknown. Here, we studied natural variation of water use in *Arabidopsis thaliana* to understand how climatic history impacts water‐use strategies. We performed a survey of vegetative water use (VWU) and life‐history traits across *A. thaliana* ecotypes in controlled and outdoor settings. Performance of select ecotypes in controlled experiments was reflective of performance in outdoor conditions. Through trait‐climate and genome‐wide association (GWAS) analyses, we tested for signals of environmental adaptation in water use. Ecotypes from warmer environments were noted in displaying enhanced water use, independent of precipitation. GWAS identified the transcription factor *MYB59* as a determiner of VWU. Functionally significant *MYB59* SNPs showed associations with temperature, but not precipitation, and *myb59* mutants demonstrated reduced water use under high temperatures. Our study suggests intraspecific variation in water‐use can be explained in part by climatic history, where temperature is the most significant driver. *MYB59* appears to contribute to this association and represents a promising candidate for future investigation in crops.

## 1 Introduction

Soil water availability is the most fundamental constraint to the net primary productivity of ecosystems (Craine et al. 2012; Zhang et al. 2017) and crop yields (Elliott et al. 2014; Lobell et al. 2020). This is reflected by the plethora of evolutionary adaptations that facilitate the acquisition and retention of water (Delwiche and Cooper 2015). Climate change is expected to alter both the quantity and temporal distribution of precipitation while simultaneously increasing temperatures and evaporative demand, thereby intensifying constraints on plant‐water relations across both natural and agricultural systems (Ali Raza et al. 2025). Understanding how plants regulate water use and how this variation has been shaped by climatic history is therefore important for predicting species response to future climates and for developing crops adapted to non‐benign environments.

The trade‐off between water loss and photosynthetic carbon gain is central to the constraining nature of water availability (Leakey et al. 2019; Brendel 2021). Thus, it follows that indices of water use efficiency (WUE) in natural systems correlate with trends in precipitation (Adams et al. 2019; DeLeo et al. 2020). Due to the lack of biochemical harnessing, the evaporation of water through stomata, that is, transpiration, has been defined as a *loss* (Morison et al. 2008; Brendel 2021), despite its importance for promoting growth via numerous physiological processes (Fricke 2017; Ferguson et al. 2021). In warm environments, transpiration is critical for facilitating leaf cooling, thereby stabilizing photosynthesis and leaf energy budgets. The degree to which transpiration or physical traits facilitate persistence in warm environments depends on water availability. However, in both hot‐wet and hot‐dry environments, transpiration has been demonstrated to be important for stabilizing leaf temperatures across multiple species (Lin et al. 2017).

The natural variation present in the model species *Arabidopsis thaliana* has been well utilized to understand the physiological and genetic mechanisms that control water‐use and water‐use efficiency (WUE). For example, integrated measures of intrinsic WUE (assessed via leaf δ^13^C) have been employed to observe a common link with phenology (McKay et al. 2003, 2008; Juenger et al. 2005) as well as to identify genetic loci and candidate genes regulating WUE (Masle et al. 2005; Des Marais et al. 2014; Campitelli et al. 2016). Recent genome‐wide association studies (GWAS) have continued to advance our understanding of WUE regulation, with natural variation approaches identifying diverse cellular processes affecting WUE, including genes involved in biomass accumulation, calcium signaling, and microtubule organization (Bhaskara et al. 2022). The extent to which climatic variation shapes selection on δ^13^C is complex (Brendel and Epron 2022), with findings in *A. thaliana* highlighting seasonal variation in water availability as a selective pressure on WUE in non‐arid environments (Dittberner et al. 2018).

Any potential associations are difficult to dissect due to the influence of confounding effects not associated with climate, such as competition, which has been shown to play an important role in this dynamic, where lines with low WUE actually perform better under increased resource competition (Campitelli et al. 2016). Despite this, directional natural selection in *A. thaliana* is strongest in arid environments (Exposito‐Alonso et al. 2019), which is reflected by the strong correlation between drought intensity and the frequency of loss of function alleles (Monroe et al. 2018). However, it is important to remember that drought resistance, whether achieved through tolerance or avoidance, is not necessarily achieved by the same mechanisms as those that regulate WUE (Lovell et al. 2013; Kenney et al. 2014; Campitelli et al. 2016; Ferguson et al. 2018; Lorts and Lasky 2020; Brendel and Epron 2022). For example, high WUE has been linked to drought sensitivity (early closing stomata), whereas low WUE can correspond to a drought escape strategy due to early flowering (Brendel and Epron 2022). These strategies clearly involve very different molecular or physiological mechanisms (Ferguson et al. 2018), especially when biomass gain, and water loss are upscaled to the whole plant level.

Climate has been successfully determined as a selective agent across *A. thaliana* natural variation, especially along altitudinal and latitudinal clines (Stinchcombe et al. 2004; Wolfe and Tonsor 2014; Vidigal et al. 2016; Tabas‐Madrid et al. 2018; Sartori et al. 2019; https://paperpile.com/c/2gnjag/dLor+DscL+kYU1+scfc), where observed trends are often described relative to flowering time. A key exception to this is the study of Vasseur et al. (2018), who observed that increasing ruderality, that is, plants that reproduce quickly and preferentially invest resources into seed production was linked with mean annual temperature at sites of origin (Vasseur et al. 2018; May et al. 2017). The manner of this association highlights how ecotypes from warmer environments operate less conservatively in terms of resource use. When considering water use, this could be hypothesized to co‐occur with increasing photosynthesis and therefore reduced WUE (Dittberner et al. 2018; Vasseur et al. 2018). Ecological shifts in *A. thaliana* have also been linked to water availability and humidity levels, suggesting that *A. thaliana* originated in dry and arid regions and adapted to high precipitation or humidity regions (Lou et al. 2022). While these studies facilitate a better understanding of how general ecological strategies transect environmental gradients, they do not explicitly test how climatic selection shapes the use of any given resource, such as nitrogen or water. It has been postulated that examining key traits, such as resource use, and the genes that underlie these traits in a global ecosystem context will be essential for resolving outstanding questions in ecology and molecular biology (Takou et al. 2019). Moreover, the applicability of such traits to crop performance may help tailor crop improvement at a regional level, since a substantial proportion of variation in breeding systems can be ascribed to climate (Ansaldi et al. 2018).

**FIGURE 1:**
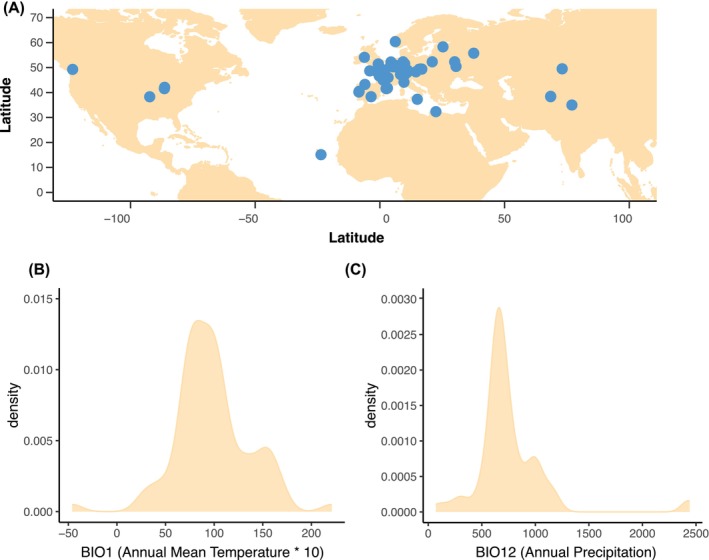
Ecotypes comprising present study. (A) Geographic point of origin of all ecotypes. (B) Variation in BIOCLIM 1 (Mean annual temperature) of all ecotypes. (C) Variation in BIOCLIM 12 (Annual precipitation) of all ecotypes.

Present understanding of how climatic history has shaped intraspecific variation in plant water use is limited but critical for understanding determinants of community dynamics and for guiding crop development for non‐benign environments. *A. thaliana* is an ideal model for this purpose due to the availability of natural ecotypes, genetic resources and the wealth of climatic data from their place of origin. For inferences towards crop design, it is essential to know about whole plant water use. Vegetative water‐use (VWU; described in Section 2) is a good proxy for this, as it encompasses whole plant traits (total leaf surface area), a major determinant of whole plant transpiration, which can overcompensate variations in stomatal conductance (Bogeat‐Triboulot et al. 2019). We have previously demonstrated the measurement/screening for water use in *A. thaliana* through short term pot drying experiments (Ferguson et al. 2018). The vegetative water use (VWU) measured via this approach reflects whole plant transpiration can be a useful tool for forecasting lifetime water‐use and we have used this to identify associated quantitative trait loci (QTL) in a biparental population (Ferguson et al. 2019). However, such artificial populations are not applicable for defining climate‐trait interactions. To this end, we focused this study on a detailed physiological characterization of VWU in a diverse panel of natural variants to explore how climatic history exerts selective pressures on water use in *A. thaliana* (Figure 1). In support of this, we integrated genome‐wide association mapping (GWAS) with transgenic manipulation and exploration of allelic variation at a VWU candidate gene to better understand the dynamic between climatic history and water use. We hypothesized that: (i) physiological performance and fitness measured under controlled conditions would be reflective of performance in more natural environments; (ii) natural variation in whole‐plant water use would have a significant genetic basis; and (iii) allelic variation underlying water use.

## 2 Materials and Methods

### 2.1 Controlled Growth Environment Experiment

Seventy‐eight *A. thaliana* ecotypes (Table S1) from the globally distributed Regional Mapping (RegMap) panel (Horton et al. 2012) were randomly selected and grown as part of the controlled growth environmental conditions experiments. Plants were grown in different temporal blocks over a period of 2 years where ecotypes Col‐0 and C24 were common to each block. 10–15 biological replicates of each ecotype were grown. Plants were initially grown in a custom walk‐in growth chamber before being transitioned to a glasshouse after the dry down experiment (see below), thereby replicating a short‐ to long‐day transition. The conditions within both growing environments were exactly as previously described (Ferguson et al. 2018, 2019).

At 40 days post sowing, plants were subjected to a period of water withdrawal from ~100% to ~20% relative soil water content (rSWC) as described in Bechtold et al. 2016. The change in rSWC for each plant was modeled by a linear model to determine vegetative water use (VWU) (Ferguson et al. 2019).

Flowering time and the number of leaves at flowering were measured as the time point (day) of bud initiation. Upon opening of the final flower, plants were bagged and allowed to dry down. Above ground biomass was separated into three components: seeds, stalks and pods, and rosette. Harvest index was calculated as the ratio of seed biomass to total above ground biomass.

### 2.2 Outdoor Experiment

Six ecotypes (C24, Col‐0, Ct‐1, Est‐1, HR‐5, and Se‐0) which were part of the original 78 ecotypes were selected based on their VWU profile for an outdoor experiment. After sowing, all seeds were stratified to promote germination. Initially, plants were grown in the above‐described short‐day controlled conditions as detailed previously (Ferguson et al. 2018, 2019). At 35 days post sowing, plants were transplanted into an outdoor planting bed (1 m × 1 m × 0.6 m) filled with peat‐based compost (Levington F2 + S, The Scotts Company, Ipswich, UK). The planting bed was located on the roof of the Life Science department at the University of Essex (51.87° N, 0.95° E). The outdoor experiment lasted 10 weeks from August 2015 until October 2015, where weather conditions did not deviate from average for this time of year (Table S4). Plants were grown following a randomized design in rows and columns 8 cm apart. Thirty plants of each ecotype were grown.

Flowering and biomass accumulation were recorded at the end of the experiment as per the controlled environment experiments. In addition, we sampled a random fully expanded upper rosette leaf 5 days post bud initiation from five biological repeats of each ecotype per treatment. The sampling was ~25 days post transplanting. Here, whole leaves were sampled immediately in liquid nitrogen to halt enzymatic activity and then used for measurements of leaf carbon content and carbon isotope composition (δ^13^C) via isotope ratio mass spectrometry as described previously (Roussel et al. 2009). δ^13^C was also determined on five biological repeats of the same ecotypes growing as part of the above‐described controlled growth conditions experiment.

### 2.3 Statistical Analysis and Figure Generation

All statistical analyses were performed within R. For all traits, we used the lme4 package to construct linear mixed models to explore sources of phenotypic variance where *Ecotype* and *Temporal block* were treated as random effects. From these, we estimated broad sense heritability (*H* ^2^) as the ratio of the ecotype variation to the total observed variation. In addition, we extracted best linear unbiased predators (BLUPs) for each ecotype and added these to population means to generate predicted means (Table S2) that controlled for the variation not due to ecotype.

We explored correlations between all traits via Pearson correlations, where *p*‐values were Bonferroni corrected. We used the corrr package to visualize all pairwise correlations as a network, where traits were spaced according to multidimensional scaling. We further explored the relationship between the phenotypic traits in all ecotypes via principal component analysis (PCA) using the FactoMineR package, where data were scaled to unit variance. Scree plots were inspected to determine significant principal components (PCs), which were defined as those that explained more variance than would be expected by chance (Kaiser 1991). To identify representative ecotypes, we first computed a distance matrix using Euclidean measures based on the entire phenotypic data set. We then used the Ward2 agglomeration to perform a hierarchical clustering analysis. The hierarchical clustering was visualized using a fan dendrogram where five groups were chosen for cutting the tree.

Climatic data from the point of origin of all ecotypes were extracted using the R package raster. This generated a dataset consisting of 18 biologically relevant climatic (BIOCLIM) variables based on latitude and longitude (Hijmans 2012). PCA was performed on the climatic dataset as described for the trait dataset. Following the methodology of (Wolfe and Tonsor 2014), we explored the correlations between significant trait PCs (TPCs) and climatic PCs (CPCs) as evidence of climatic selection on phenotypic variance. This was achieved by performing Pearson's product moment correlation testing between each TPC‐CPC pairwise interaction.

Simple linear statistical models were constructed to test for interactions between traits that were measured in common between the controlled environment experiment and the outdoor experiment.

All figures were produced within R using base plotting functions and ggplot2. Additional post‐processing was performed in Affinity Designer2.6.4.

### 2.4 GWAS and Population Genetics Analysis

We used the R package adegenet (Jombart 2008) to group ecotypes based on genetic similarity using the 250 k RegMap single nucleotide polymorphism (SNP) dataset (Horton et al. 2012). Initially, the find.clusters() function was used to transform the SNP matrix via PCA before running iterative *K*‐means clustering. The most appropriate clustering model was selected according to the Bayesian information criterion (BIC). Subsequently, the dapc() function was used to perform discriminant analysis of principal components (DPAC; Jombart et al. 2010) to define the diversity between the pre‐identified groups. We performed Kruskal–Wallis rank sum tests to test for significant differences in mean annual temperature and VWU between the identified DAPC groups.

Genome‐wide association mapping (GWAS) was performed for VWU with GWAPP (Seren et al. 2012), which uses ~206,000 SNPs generated from ecotypes that were genotyped for 214,000 SNPs and/or sequenced via next generation sequencing. We acknowledge that incorporating < 100 populations into GWAS analyses is typically cautioned against due to reduced statistical power. However, given instances where population number is restricted it is still possible to associate genetic and phenotypic variation, especially when trait variation is substantial (Lehnert et al. 2018; Soumya et al. 2021). To this end we note the success of other Arabidopsis studies that have incorporated low numbers of populations (70–150, Zou et al. 2017; de Ollas et al. 2023; Wang et al. 2024). In addition, we have carried out GWAS according to best practices, used a stringent significance criterion for identifying trait associated SNPs and performed validation on a candidate gene. The accelerated mixed model (AMM) genome scan approach (Zhang et al. 2010) was used for our GWAS. We corrected the *p*‐values according to (Benjamini and Hochberg 1995) to identify SNPs below a 5% false discovery threshold. The linkage disequilibrium (LD) block (*r* ^2^ > 0.3) containing the significant SNPs was investigated to identify candidate genes using GWAPP.

AT5G59780 (*MYB59*) was identified as a candidate gene for VWU based on GWAS results. To validate the effect of *MYB59* on VWU, we generated haplotypes of *MYB59* based on the seven SNPs from the 250 K RegMap dataset (Horton et al. 2012; Table S6) that fall within the coordinates of *MYB59*. Significant differences in representative trait and BIOCLIM parameters between the two main haplotype groups were tested by fitting a one‐way ANOVA.

To test how natural allelic variation in *MYB59* was associated with temperature variation we employed the approach of (Ferrero‐Serrano and Assmann 2019) to test for associations between genetic variants and local environment. Here, we used the Polymorph variant browser (www.1001genome.org/tools) to search for SNPs with a minor allele frequency > 5% within *MYB59* with a predicted “moderate” or greater impact on the translated amino acid sequence. Subsequently, we downloaded the *MYB59* sequence data for all ecotypes from the 1001 genome initiative and filtered for those pre‐identified SNPs. These SNPs were visualized on the *MYB59* gene model using the R package genemodel (https://github.com/greymonroe/genemodel). We tested the probability density of each allele of these SNPs against variation in the BIOCLIM parameters for all ecotypes comprising the 1001 genome initiative. We omitted sequences of ecotypes from North America and the British Isles to avoid confounding effects due to recent dispersal that have not allowed enough time for selection to have occurred (Platt et al. 2010). Observed differences were tested for significance via two‐sample Wilcoxon tests.

### 2.5 *myb59* Knockout Experiments

Based on the GWAS results, AT5G9780 (*MYB59*) was selected for experimental investigation. Two previously published myb59 knockouts (*myb59‐1*, *myb59‐2*; Fasani et al. 2019; Du et al. 2019) were obtained from Drs Wang and Furini. Mutants were checked for the presence of the T‐DNA insertion (myb59‐1) and MYB59 expression using RT‐PCR (Primers: myb_fw2: GGGCGGTCTTTGGAAAGGACC and myb_rev2 CTAAAGGCGACCACTACCATG). The *myb59‐2* allele was generated using CRISPR/Cas9 and maintains a full‐length transcript; however, phenotypically, the mutant copies the *myb59‐1* mutant, suggesting that the protein is not functional (Du et al. 2019).

Seeds from both knockouts and Col‐0 wildtype were sown as described above and short dehydration experiments were carried out at 22°C (day/night) and 27°C/22°C (day/night) short‐day conditions to determine VWU. Water potential was measured using a WP4C Water Potential Meter (Labcell Ltd., UK) on leaf disks from 6 independent plants at the mid‐point of the light period. For stomatal densities, the abaxial side of the leaf was painted with clear nail varnish and the peels were analyzed for stomatal densities using a Zeiss Axioskop 20× magnification connected to a Retiga 2000R camera. Additionally, to the above‐described experiment, Col‐0, *myb59‐1* and *myb59‐2* alleles mutants were sown on ½ MS media to assess root length at 22°C (day/night) and 27°C/22°C (day/night) conditions. Roots were measured using ImageJ at every 2 days post germination up until 2 weeks after sowing. Leaf temperature was measured using an infrared thermal imaging camera (HIKMIKRO) before the start of the VWU measurements.

## 3 Results

### 3.1 Extensive Natural Variation in Vegetative Water Use, Phenology, and Biomass Accumulation

To study the natural variation of water‐use in *A. thaliana* we subjected 78 ecotypes (Figure 1, Table S1) to a period of water withdrawal as described previously (Ferguson et al. 2018, 2019). We have previously shown that this water withdrawal period does not elicit a classic “drought escape” floral transitioning mechanism, nor does it reduce subsequent vegetative or reproductive biomass (Ferguson et al. 2018).

**FIGURE 2:**
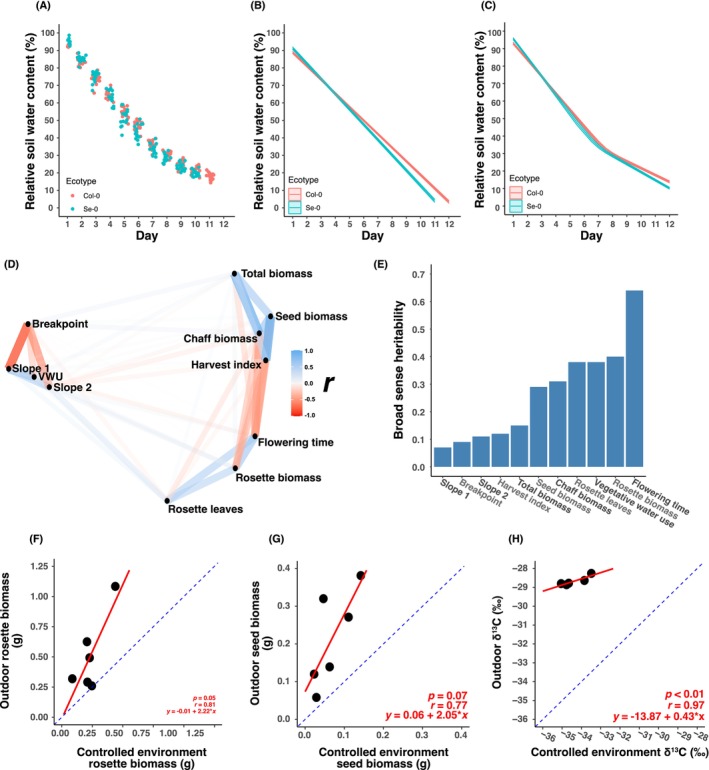
Modeling water use and trait correlations. (A) Relative soil water content (rSWC) of Col‐0 and Se‐0 ecotypes during the dry‐down period (*N* = 15). (B) Linear model of rSWC as a function of elapsed time in days. Vegetative water use (VWU) is defined as the slope of the linear model. (C) Segmented model of rSWC as function of elapsed time in days. (D) Network plot showing the relationship between all traits measured as part of the controlled experiment. The width of the band connecting each trait is proportional to the *p*‐value and the color of the band is associated to the correlation coefficient (*r*). The *p*‐values and *r* values can be found in Table S2. (E) Broad sense heritabilities (*H* ^2^) of all traits measured. (F–H) Correlation between vegetative biomass (F), reproductive biomass (G) and carbon isotope composition (H) measured in the controlled and outdoor experiments. For each plot the linear model (red, solid) and one‐to‐one line (blue, dashed) are provided. The *p*‐value, *r*, and linear equation are inset in each plot.

Although the response of relative soil water content (rSWC) to elapsed time was marginally curvilinear (e.g., Col‐0 and Se‐0 as shown in Figure 2A), we modeled the overall response as a linear regression (Figure S1), and the associated slope provides an estimate of vegetative water use (VWU) over the duration of the dry down period (Figure 2B; Ferguson et al. 2018). We also modeled the response of VWU to drying through a segmented approach (Figure 2C, Figure S1), which provides an additional estimate of drought sensitivity (Muggeo and Attanasio 2011, Ferguson et al. 2018). Importantly, the parameters estimated from this approach (initial slope (Slope 1), breakpoint, and the secondary slope (Slope 2)) were all highly correlated to VWU (Figure 2D). Given these strong calculations, we used VWU estimated from the linear model for all subsequent genetic analyses, as it provided a parsimonious summary of water‐use across the entire dry‐down period. VWU demonstrated a 2.5‐fold variation across the surveyed diversity (Figure S2, Table S2) and moderate *H* ^2^ (Figure 2E), suggesting a discernible genetic component to the observed variation.

Across the diversity surveyed, we observed substantial variation in phenology (Figure S2, Table S2). Flowering time ranged from 45 to 105 days (Figure S2, Table S2) and demonstrated the highest *H* ^2^ of all traits comprising this study (Figure 2E). Life‐history traits such as rosette biomass, seed biomass, harvest index, and total above ground biomass also showed substantial natural variation (Figure S2, Table S2) and demonstrated low‐to‐moderate *H* ^2^ (Figure 2E).

Using data obtained from our controlled environment study (Table S2), we explored associations between all measured traits via concurrent multidimensional clustering and Pearson product moment correlation tests (Figure 2D, Table S3). Traits related to water use, biomass accumulation, and phenology were observed to independently cluster together (Figure 2D). Traits relating to water use, that is, VWU, Slope 1, and Slope 2, were positively correlated with each other and negatively correlated with the breakpoint estimated via the segmented modeling approach (Figure 1D, Table S3). VWU did not demonstrate a significant association with any further traits (Figure 2D, Table S3). This suggests that short‐term water use was not driven by development or eventual biomass accumulation. However, across a separate experiment comprising 48 ecotypes in the same controlled conditions, we did observe a significant positive association between rosette area (measured prior to the drying period used to estimate VWU) and VWU (Figure S3) which is line with previous findings (de Ollas et al. 2023). The independence of VWU from flowering time and reproductive traits suggest that variation in water use represents a distinct physiological axis rather than a consequence of life‐history variation, thereby facilitating subsequent analyses aimed at identifying a its genetic and climatic determinants.

We observed a trade‐off between time to reproduction and reproductive output (Figure 2D, Table S3). Flowering time was strongly, negatively correlated with the mass of chaff (stalks and pods) and seed biomass (Figure 2D, Table S3). This suggests that in this environment, ecotypes that delay floral transitioning have reduced fitness. Conversely, later flowering ecotypes tended to have much larger vegetative rosettes, highlighting a floral‐mediated trade‐off between vegetative and reproductive growth (Figure 2D, Table S3).

### 3.2 Comparison of Life‐History Traits Measured in Controlled Growth Room Versus Common Garden Environments

A major aim of this study was to test for phenotype‐climate associations; we assessed whether plant performance in our controlled growth room experiments was reflective of performance in a *more natural* outdoor experiment. For this, we grew a set of six ecotypes (C24, Col‐0, Ct‐1, Est‐1, HR‐5, and Se‐0) in both environments.

The performance of these ecotypes was determined via the accumulation of biomass (above ground vegetative and reproductive biomass) and via integrated water use efficiency (measured as leaf carbon isotope discrimination (δ^13^C)). In all three cases, the performance of ecotypes in controlled and outdoor environments was positively correlated (Figure 2F–H), highlighting that life‐history and water use traits measured in controlled environments can be reflective of some traits phenotyped in more natural settings.

Taken together, these comparative analyses support our first hypothesis that physiological performance measured under controlled conditions is reflective of performance in more natural environments. The positive associations between environments for biomass accumulation and δ^13^C indicate that controlled‐environment phenotyping captures ecologically relevant variation among ecotypes. This validates the use of controlled‐environment assays for testing associations between climatic history and water‐use traits across the wider diversity panel. Additionally, these results demonstrated that the outdoor environment was favorable for the ecotypes tested here, since vegetative and reproductive biomass was generally enhanced relative to controlled conditions (Figure 2F,G).

### 3.3 Associations Between Climatic Variation and Phenotypic Variation

To test for trait‐climate associations we adopted the comparative principal component analyses (PCA) approach utilized by Wolfe and Tonsor (2014), where trait principal components (TPCs) are compared to climate principal components (CPCs). It allows us to extract critical information from a complex set of phenotypic traits by reducing a larger set of initially correlated variables to a much smaller set of uncorrelated variables, while preserving information.

**FIGURE 3:**
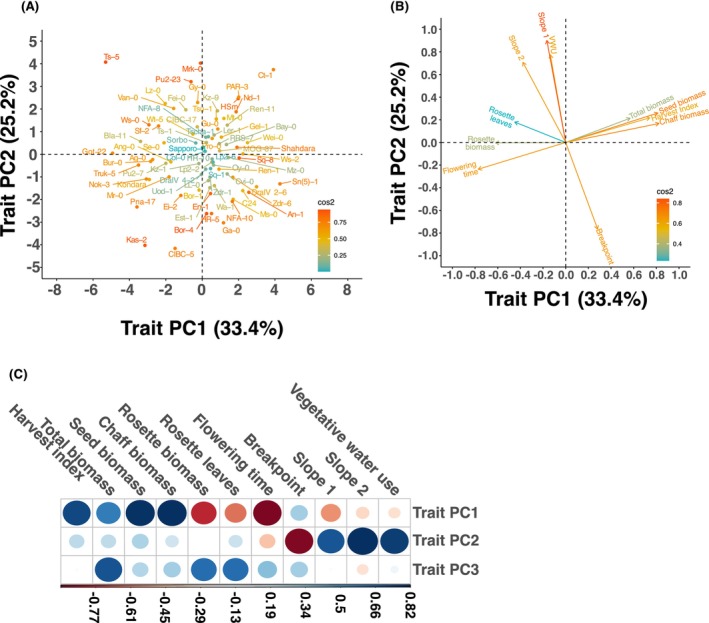
Trait principal component analysis. (A) The loading of all traits onto TPC1 and TPC2. Traits are colored according to the quality of the factor map (squared cosine (cos^2^)). (B) The association between all ecotypes with TPC1 and TPC2. Ecotypes are colored according to cos^2^. (C) Relationship between all traits with the first three TPCs. The size of each bubble is proportional to the strength of the association (*p*‐value) and the color represents the direction of the association (Pearson correlation coefficient (*r*)) as indicated by the color bar. Non‐significant trait‐PC correlations (*p* > 0.05) are indicated by the absence of a bubble.

The trait PCA (Figure 3) confirmed previous observations regarding the associations of water use, phenology and biomass accumulation associated traits (Figure 2D, Table S3). The first three TPCs were observed to explain most of the observed variation (73.7%; Figure S4A). TPC1 explained 33.4% of the total trait variation and was predominantly associated with phenology (negative correlation) and biomass accumulation (positive correlation for reproductive and total biomass and negative correlation for rosette biomass) (Figure 3C). TPC2 explained 25.2% of the total trait variation and was predominantly associated with water use (positive correlation) and drought sensitivity (i.e., breakpoint, negative correlation) (Figure 3C). TPC3 was characterized by positive associations with all biomass accumulation traits (Figure 3C). Importantly, the emergence of water‐use traits as an independent principal component justified their subsequent comparison with climatic variation, allowing us to directly test whether climatic history has shaped natural variation in water‐use.

**FIGURE 4:**
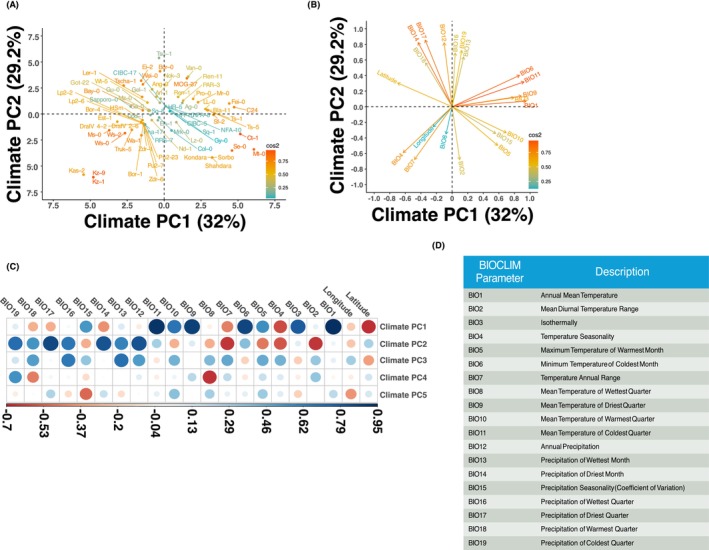
Climate principal component analysis. (A) The loading of all traits onto CPC1 and CPC2. Climate parameters are colored according to the quality of the factor map (squared cosine (cos^2^)). (B) The association between all ecotypes with CPC1 and CPC2. Ecotypes are colored according to cos^2^ (C) Relationship between all traits with the first five CPCs. The size of each bubble is proportional to the strength of the association (*p*‐value), and the color represents the direction of the association (Pearson correlation coefficient (*r*)) as indicated by the color bar. Non‐significant trait‐PC correlations (*p* > 0.05) are indicated by the absence of a bubble. (D) Definition of BIOCLIM parameters used in the CPCA.

For the climate PCA (Figure 4), we observed that the parameters related to precipitation, temperature, and seasonality tended to cluster with one another, with a few exceptions (e.g., BIO8 and BIO2; Figure 4A). The first five CPCs explained most of the total climatic variation (90.1%, Figure S4B). CPC1 explained 32% of the total variance and was most strongly associated with temperature (positive correlation) and temperature seasonality (negative correlation) (Figure 4A–C). A key for the BIOCLIM parameters is shown in Figure 4D.

**FIGURE 5:**
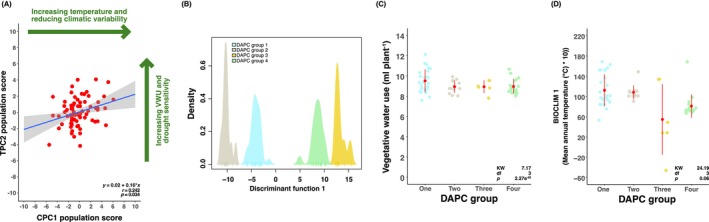
Association between temperature and water use. (A) Correlation between the ecotype population score for climate principal component 1 (CPC1 population score) and the ecotype population score for trait principal component 2 (TPC2 population score). The linear regression of the relationship (blue) and standard error of the model (gray) are plotted. The correlation coefficient (*r*) and associated *p*‐value cut‐off are inset. The most important parameters associated with CPC1 and TPC2 are highlighted in green to aid interpretation. (B) Summary of discrimination analysis of principal components (DAPC) to group ecotypes based on genetic similarity. Each tick on the discrimination function 1 axis represents an individual ecotype. Each cluster of ecotypes is named and colored uniquely. (C, D) Vegetative water use and mean annual temperature of all ecotypes grouped according to their DAPC classification. Kruskal–Wallis test statistics are inset. Red points and error bars denote the DAPC group mean and standard deviation.

To look for signals of climatic selection on plant performance, we subsequently tested for associations between the significant TPCs and CPCs (i.e., Table S4) following the approach of Wolfe and Tonsor (2014). Of the 15 possible association tested here, we only observed a significant association between CPC1 and TPC2, where the association was positive (Figure 5A). This association suggest that ecotypes exhibiting increased VWU tend to originate from environments characterized by warmer temperatures and reduced temperature seasonality. Although modest, this relationship is consistent with temperature contributing to natural variation in water‐use.

We additionally clustered the ecotypes according to genetic similarity via discrimination analyses (Figure 5B). Comparing VWU and BIOCLIM 1 (mean annual temperature) across the distinct genetic ecotype clusters demonstrated that both parameters were significantly different across these groups (Figure 5C,D). Moreover, the two groups that clustered negatively (DAPC group one and two) onto the first discrimination function (which explains most of the genetic variation) demonstrated higher VWU (Figure 5C) and were from warmer environments (Figure 5D) compared to the two groups that clustered positively (DAPC group three and four) onto this first discrimination function. Together, these findings suggest that the observed association between temperature and VWU is underpinned by genetic variation among ecotypes, providing further rationale for identifying the loci contributing to natural variation in water use, as described below.

### 3.4 Identification of *MYB59* as a Candidate Gene for Vegetative Water Use

**FIGURE 6:**
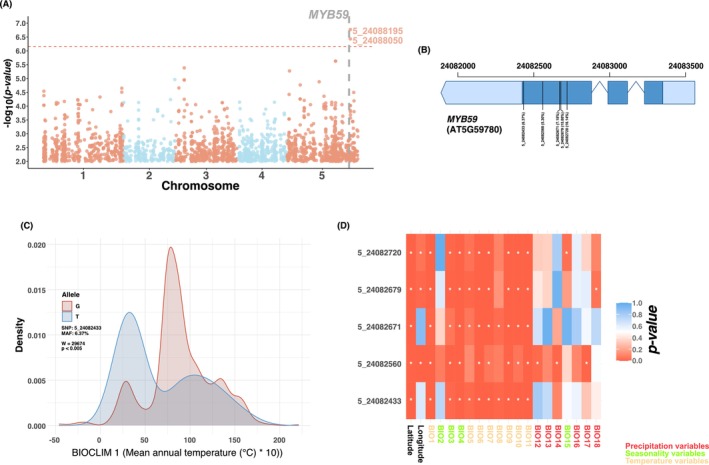
GWAS for vegetative water use (VWU) and exploration of allelic variation at *MYB59*. (A) Manhattan plot showing the results of GWAS for VWU. A suggestive cut‐off 6 −log_10_(*p*‐value) is highlighted, where the two SNPs above this threshold have FDR below 0.05. The position of *MYB59* is indicated. (B) *MYB59* gene model with position on chromosome 5 indicated. Light blue regions indicate untranslated regions. Dark blue regions indicate exons. Thin blue lines indicate introns. The location of SNPs with predicted modifying effects on the protein structure are highlighted. (C) Probability density of the two alleles of SNP 5_24082433 against BIOCLIM parameter 1. The statistics of a two‐paired Wilcoxon test are inset. (D) Heat map showing the *p*‐values from two‐paired Wilcoxon tests where allelic variation at each of the *MYB59* functional SNPs is tested against latitude, longitude and the 18 BIOCLIM parameters. The BIOCLIM parameters are colored according to whether they represent temperature, precipitation, or seasonality variables.

We performed GWAS to identify SNPs significantly associated with the variation observed for VWU (Figure 6A). The AMM GWAS approach identified two significant SNPs in close proximity to one another. These SNPs were at base‐pair position 24,088,050 (−log_10_(*p*‐value) = 6.77) and 24,088,195 (−log_10_(*p*‐value) = 6.43) on chromosome five (Figure 6A, Table S5). Within the LD block (*R* ^2^ > 0.3) containing the two significant SNPs, there was just a single gene, transcription factor *MYB59* (AT5G59780; base‐pair coordinates: 24,081,922–24,083,562; Figure S5).

The two major haplotypes of *MYB59* (Table S6) showed significant differences in VWU (Figure S6A), supporting our assertion of *MYB59* as a candidate gene for regulating VWU. These analyses were performed to determine whether occurring allelic variation at *MYB59* contributes to climate‐associated variation in VWU, thereby directly testing our hypothesis that genes underlying water‐use variation are associated with climatic conditions at the accession site of origin. Furthermore, these haplotype groups did not significantly differ in rosette biomass (Figure S6B) or flowering time (Figure S6C), thereby suggesting that the variance in VWU imparted by *MYB59* was not linked to any impact of *MYB59* on phenology or overall plant size. The only other parameter that demonstrated a difference was Slope 1, which represents the initial portion of the dry down period (Table S6). Additionally, we observed that the mean annual temperature (BIOCLIM 1), maximum temperature of the warmest months (BIOCLIM5) and mean temperature of the warmest quarter (BIOCLIM10) at the point of ecotype origin was significantly different between the two haplotype groups (Figure S6D, Table S6). Neither mean annual precipitation (BIOCLIM 12) nor any other precipitation parameters (BIOCLIM 13–18) were significantly different between the two main haplotype groups (Figure S6E, Table S6). This highlights how temperature, but not precipitation, may exert a selective pressure on the allelic form of *MYB59*.

To test the link between climate and *MYB59* variation we queried the Polymorph 1001 genomes database for SNPs with moderate‐to‐high modifying impacts on the translated amino acid sequence of *MYB59*. Through this approach we identified multiple SNPs, all of which were defined as having moderate modifying impacts on the amino acid sequence. Five of these SNPs had a minor allele frequency (MAF) greater than 5%, which ranged from 5.30%–16.14% (Figure 6B). We tested for associations between allelic frequency of these SNPs and climate at ecotype point of origin. The missense variant of the SNP at base‐pair position 24,082,433, for example, was observed to cluster in cooler environments (Figure 6C). In general, when comparing allelic frequency of all five SNPs against all 18 BIOCLIM parameters and latitude and longitude, we noted that significant differences in the probability densities of associated alleles were restricted to temperature variables, including seasonality (Figure 6D), suggesting that temperature, and not precipitation or climate variability, exerts selective pressure on the allelic form of *MYB59*, providing strong support for our hypothesis that genes underlying water‐use variation are associated with temperature at the point of origin.

### 3.5 *MYB59* Affects Water Use Under Elevated Temperature Conditions by Altering Root Length and Stomatal Densities

**FIGURE 7:**
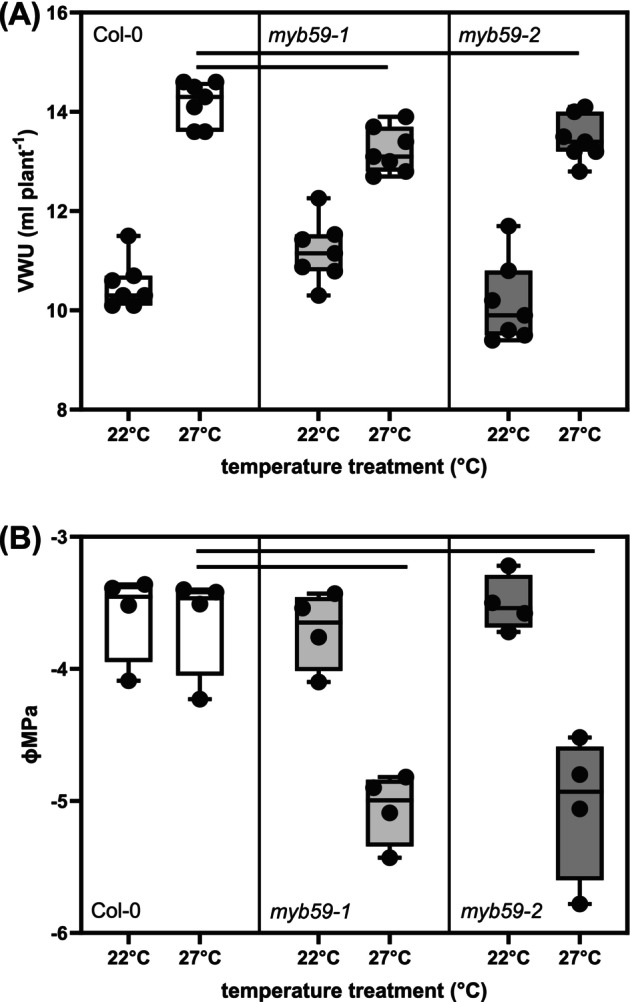
The role of *MYB59* on water use‐associated traits. (A) Vegetative water use (VWU) of wildtype and *myb59* knockout lines under control and warm temperatures. (B) Leaf water potential of wildtype and *myb59* knockout lines under control and warm temperatures. Horizontal lines indicate significant difference compared to Col‐0 (A) Col‐0 versus *myb59‐1 p* < 0.0025; Col‐0 vs. *myb59‐2 p* = 0.019 and (B) Col‐0 vs. *myb59‐1 p* = 0.0025; Col‐0 vs. *myb59‐2 p* = 0.0027 (one‐way ANOVA, posthoc Tukey, *n* = 4).

To test the importance of *MYB59* for regulating water use in warm environments we utilized two independent *myb59* knockout lines alongside wildtype. Here, we phenotyped VWU under control (22°C) and warm (27°C) conditions. In general, VWU increased under the warm conditions relative to the control treatment (Figure 7A). However, VWU was significantly reduced in the *myb59* knockout lines relative to the wildtype under the warm conditions, but not the control conditions (Figure 7A). These differences could have been due to the marginally but not significantly increase in rosette areas between Col‐0 and both *myb59* alleles (Figure S7). We also observed that the *myb59* knockout lines demonstrated significantly reduced leaf water potential under warm conditions (Figure 7B), which is indicative of perturbed water use and water status in these lines relative to wildtype.

Importantly, stomatal density, which is known to be influenced by *myb59* (Fasani et al. 2019), showed a significant reduction at both temperature treatment (Figure S8), yet VWU was only reduced under warm temperatures (Figure 7A), suggesting that differences in stomatal patterning and/or behavior are unlikely to underlie the observed differences in VWU under warm temperatures. Often, a change in stomatal density is accompanied by a change in cell size, which may not change the overall transpiration capacity.

Since water use is also a function of root architecture and *MYB59* is known to influence root traits (Du et al. 2019), we additionally profiled root length of the two *myb59* knockouts and wildtype under the elevated temperature treatment. Root length in the *myb59* knockout lines was increased when the plants were growing under the warm treatment (Figure S9). The difference in root length between the *myb59* knockout lines and wildtype was further reflected by differences in root biomass and the root:shoot ratio (Figure S10).

## 4 Discussion

Classical evidence from crop species has highlighted how artificial selection in warm environments has inadvertently selected for heat avoidance via transpiration (Radin et al. 1994; Koester et al. 2016). Despite this, little evidence exists to determine which selective agents define intraspecific variation of water use in natural populations. In this study, we explored natural variation of VWU in *A. thaliana* and tested for associations between this variation and climatic variation at the site of origin of the ecotypes comprising the study. Overall, our findings support the three hypotheses proposed in this study. First, physiological performance measured under controlled conditions was broadly reflective of performance in a more natural outdoor environment, validating the use of controlled‐environment phenotyping to investigate ecologically relevant variation in water‐use. Second, VWU exhibited substantial heritable variation and genome‐wide association mapping identified MYB59 as a candidate regulator, demonstrating a significant genetic basis to natural variation in whole‐plant water use. Third, both trait‐climate analyses and the geographic distribution of *MYB59* alleles indicated that temperature, rather than precipitation, is an important selective agent shaping water‐use strategies in *A. thaliana* .

The variation in VWU observed was heritable (Figure 2E) and the response of relative soil water content during the short‐dehydration experiments appears coordinated to known transcriptomic responses with respect to the breakpoint in the relationship between rSWC and time and known changes in gene expression during the same experimental dry down period (Bechtold et al. 2016). Taken together, these findings highlight a strong genetic component to VWU variation in *A. thaliana* . This dataset builds upon a large body of work that describes natural variation of traits that underpin water use in *A. thaliana* (Dittberner et al. 2018; Ogura et al. 2019; Bhaskara et al. 2022; Elfarargi et al. 2023), by measuring water use across an extended vegetative period.

Across the ecotypes comprising this study, we observed the classical trade‐off between investment in vegetative biomass accumulation and reproductive output (Figure 2D) that is well‐understood to be mediated by flowering time (Ågren et al. 2013; Ellis et al. 2021). Biomass accumulation associated to the vegetative rosette is a good approximation of plant size (Ferguson et al. 2018) and we observed significant variation across the surveyed ecotypes to this end (Figure 2E, Figure S2). Plant size is often associated with enhanced water use (Feldman et al. 2018), however in our study we did not observe a significant association between rosette biomass and VWU or any of the other water use‐associated traits (Figure 2D). This is an important observation for this study, as it suggests that VWU variation is not a function of size. Therefore, any genetic or climatic association with VWU are unlikely to be a function of variation in flowering time. This is important because flowering time is under strong selection to adapt populations to local climate (Beckerman et al. 2002; Ågren et al. 2017) and is therefore often associated with climatic variation (Stinchcombe et al. 2005). Consequently, the independence of VWU from phenological variation here guards against the identification of spurious association between VWU and climate parameters or SNP markers that may otherwise have had arisen had VWU been linked to biomass accumulation.

Most of our understanding on plant growth and development in *A. thaliana* has come from controlled environments. Here, we have shown that the performance of ecotypes growing in our controlled environment studies matches up with performance from more natural settings (Figure 2F–H). These rank correlations between controlled and outdoor environments are supportive of exploring associations between phenotypic variation assayed in controlled environments and climatic variation at the point of origin of ecotypes. Previous work in this area has produced evidence highlighting climatic selection on traits that underpin growth and development. Through comparing TPCs and CPCs Wolfe and Tonsor (2014) demonstrated selection for increased investment in vegetative growth with reduced temperature and increased precipitation across 48 ecotypes that span a relatively small altitudinal gradient in north‐eastern Spain. Similarly, Dittberner et al. (2018) directly compared traits with environmental PCs to show associations between WUE and stomatal characteristics; most notably, they demonstrated a positive association between stomatal size and temperature. Through comparing climate‐trait principal components and corroborating those results with differences in climate and trait parameters between genetic clusters of ecotypes, our study suggests that temperature may contribute to shaping natural variation in VWU (Figure 5). This finding is in accordance with reciprocal transfer experiments that have demonstrated a developmental switch to enhanced water use to promote cooling capacity in *A. thaliana* (Crawford et al. 2012).

*A. thaliana* is particularly sensitive to temperature changes (Ågren and Schemske 2012; Clauw et al. 2022). Due to its influence on growth, temperature has been demonstrated to be key in determining the distribution of *A. thaliana* (Ågren and Schemske 2012; Clauw et al. 2022). Moderately elevated temperatures that are below heat shock (27°C), as used in our experiments incorporating *myb59* mutants, do not trigger immediate stress responses, but may alter plant growth and development, for example, root elongation, petiole hyponasty, and reduced stomatal index (Lee et al. 2021; Ludwig et al. 2021). Root elongation and transpiration rate increases in response to high temperatures regardless of watering conditions, highlighting the importance of a latent cooling capacity. Warm temperatures affect roots directly and indirectly, via decreases in shoot carbon availability and changes to root water relations due to increased transpirational demand, all of which can affect growth, water‐, and nutrient uptake (Calleja‐Cabrera et al. 2020). Root biomass and stomatal density have both been linked to temperature dependent acclimation of *A. thaliana* ecotypes. Ecotypes originating from sites with warmer temperatures allocated less biomass to the roots but showed a significant positive effect on reproductive allocation when grown under high temperatures (Vile et al. 2012). Here, local adaptations to warm environments appeared to be driven at the leaf level through reduced stomatal density, and via reduced biomass allocation to roots compared to ecotypes from colder sites when cultivated under high temperatures (Vile et al. 2012). On the other hand, high temperature environments have been shown to result in longer primary root length in exploration for water (Martins et al. 2017). We did not observe a significant difference in root length in wildtype plants grown under 22°C or 27°C, which may reflect the readily available water of plate grown plants (Figure S10).

Given the context of the above‐described trade‐off between root elongation to facilitate water uptake for evaporative cooling and water conservation, the identification of *MYB59* via GWAS for VWU was especially interesting as this transcription factor is involved in cell cycle progression and root growth (Mu et al. 2009; Fasani et al. 2019). *MYB59* is expressed at higher levels in the roots specifically in xylem cells and might be involved in the transcriptional regulation of xylem differentiation (Oh et al. 2003). Over‐expression of *MYB59* represses root growth (Mu et al. 2009; Enomoto et al. 2023), and conversely, a *myb59* knockout mutant showed enhanced root elongation when grown on MS media containing 1% sucrose (Fasani et al. 2019).

In our study, *myb59* mutants used significantly less water and had lower water potential under the warm temperatures compared to wild type (Figure 7), which is likely due to a collation of factors including altered root growth and reduced stomatal density, all of which may contribute to improved water homeostasis under elevated temperature (Figures [Link], [Link]). The long root phenotype at elevated temperatures in *myb59* mutants is, however, counterintuitive to the reduction in VWU under elevated temperatures. Importantly, this observation does not contradict the proposed role of *MYB59* in water‐use regulations but instead highlights that whole‐plant water‐use emerges from interactions among multiple traits, whole relative importance may vary across environments.

*MYB59* also plays a role in the balance of plant nutrient levels especially potassium (K^+^) and nitrate (${\mathrm{NO}}_{3}^{-}$). Under low K^+^/${\mathrm{NO}}_{3}^{-}$, the alternative splice variant MYB59α regulates the induction of the K^+^/${\mathrm{NO}}_{3}^{-}$ symporter NPF7.3, enhancing root‐to‐shoot translocation of K^+^ and over‐expression of the *MYB59α* variant suppresses root growth under K^+^ replete conditions (Enomoto et al. 2023). Conversely, *myb59* mutants were shown to be defective in K^+^/${\mathrm{NO}}_{3}^{-}$ root‐to‐shoot translocation when grown on high levels of K^+^/${\mathrm{NO}}_{3}^{-}$, and under those conditions plants also exhibited an elongated root phenotype (Du et al. 2019).

Recent work has further revealed that the MYB59‐NPF7.3 regulatory module is under diurnal control, with HY5 and PIF transcription factors alternately modulating xylem K^+^/${\mathrm{NO}}_{3}^{-}$ loading in response to light–dark cycles (Jing et al. 2025). This integration of light signaling with nutrient transport regulation highlights the multifunctional nature of MYB59 in integrating plant responses to multiple environmental stimuli. K^+^ movements are linked to water transport and accumulation of K^+^ in the vacuole allows plants to adjust water flux and cell water potential under water‐limiting conditions, and therefore a defect in K^+^ transport may limit water movements despite the long root phenotype.

Furthermore, experiments to determine root length were carried out on plates under well‐watered and high humidity conditions, while water use was determined in soil grown plants subjected to short‐term dehydration and elevated temperatures. While root functional traits strongly influence water‐use and nutrient uptake, plants may shift allocation among tissues to acquire resources that most limit growth depending on the environmental context, that is, the need to conserve water (during short‐term dehydration combined with elevated temperature), or the need for evaporative cooling (long term elevation of temperature).

## 5 Conclusion

Our results suggest that *MYB59* contributes to subtle root and stomatal variation associated with warmer environments (Figures 6 and 7, Figures [Link], [Link]), but not necessarily short‐term stress responses to elevated temperatures, and under cooler temperatures these differences were largely not significant. The temperature‐dependent manner with which this trait variation is expressed highlights the complexity of the mechanisms underlying whole‐plant water‐use. Functional variation at *MYB59* may contribute to adaptation to warm environments through its influence on traits linked to water use and evaporative cooling and balancing nutrient and water requirements under prevailing environmental conditions. This is reflected in *MYB59* alleles demonstrating differential probability densities across temperature‐associated bioclimatic parameters, such as mean annual temperature. This same effect was not observed with precipitation‐associated parameters, which is consistent with MYB59 contributing to temperature‐associated variation in water‐use (Figure 5). The identification of MYB59 as a temperature‐responsive regulator of water use is consistent with emerging evidence that MYB transcription factors serve as central integrators of environmental signals, coordinating responses to temperature, light, and nutrient availability (Jing et al. 2025; He et al. 2023). This multifunctional capacity may be particularly important for adaptation to warm environments where plants must balance competing demands for evaporative cooling, nutrient acquisition, and water conservation.

## Funding

J.N.F. was supported by a BBSRC‐CASE studentship (BB/J012564/1). The SILVATECH facility is supported by the French National Research Agency through the Laboratory of Excellence ARBRE (ANR‐11‐LABX‐0002‐01).

## Conflicts of Interest

The authors declare no conflicts of interest.

## Supporting information


**Figure S1:** Comparison of *R* ^2^ between linear models and segmented models. Boxplots describe the spread in *r* ^2^ of linear and segmented models describing vegetive water‐use (VWU) as a function of days post watering of all plants comprising the VWU ecotype screen.

**Figure S2:** Histograms showing the variation in predicted means for all ecotypes and all traits measured as part of controlled environment experiment.

**Figure S3:** Correlation of total rosette area and vegetative water use (VWU) from the separate experiment. The blue line represents a linear model scribed VWU as a function of rosette area. The *p*‐value and *R* ^2^ values from the linear model are inset.

**Figure S4:** Scree plots describing the amount of variation of the total trait and climate variation that is described by each incremental principal component for the trait principal component (TPCA, (A)) and climate principal component analysis (CPCA, (B)).

**Figure S5:** Linkage disequilibrium block containing AT5G59780 (*MYB59*) and the two significant SNPs from GWAS for vegetative water‐use (VWU).

**Figure S6:** *MYB59* haplotype analysis. Boxplots describing differences in (A) vegetative water use (VWU), (B) dried rosette biomass, (C) flowering time, (D) BIOCLIM 1 (mean annual temperature) and (E) BIOCLIM 2 (mean annual precipitation) for all accessions across the two main *MYB59* haplotypes. *p*‐values associated within a one‐way ANOVA are inset.

**Figure S7:** Rosette areas of wildtype and *myb59* knockout lines. There was no significant difference between genotypes (one‐way ANOVA, *p* > 0.05, *n* = 9).

**Figure S8:** Stomatal density of wildtype and *myb59* knockout lines. Significant differences between genotypes at different temperatures were assessed using a one‐way ANOVA and a Tukey HSD post hoc test. Groups labeled with the same letter are not significantly different from one another; groups labeled with different letters are significantly different (*p* < 0.05, *n* = 20 at a minimum). Groups sharing a letter with more than one group are not significantly different from either of those groups, even where those groups differ significantly from each other.

**Figure S9:** Root length of wildtype and *myb59* knockout lines 12 days post sowing. Significant differences between genotypes at different temperatures were assessed using a one‐way ANOVA and a Tukey HSD post hoc test. Groups labeled with the same letter are not significantly different from one another; groups labeled with different letters are significantly different (*p* < 0.05, *n* = 20 at a minimum). Groups sharing a letter with more than one group are not significantly different from either of those groups, even where those groups differ significantly from each other.

**Figure S10:** (A) Root biomass and (B) root: shoot ratio of wildtype and *myb59* knockout lines grown at 27°C. Significant differences between genotypes at different temperatures were assessed using a one‐way ANOVA and a Tukey HSD post hoc test. Groups labeled with the same letter are not significantly different from one another; groups labeled with different letters are significantly different (*p* < 0.05, *n* = 7 at a minimum). Groups sharing a letter with more than one group are not significantly different from either of those groups, even where those groups differ significantly from each other.

**Table S1:** Ecotypes comprising study.
**Table S2:** Predicted means for all traits.
**Table S3:** Correlations of traits measured as part of the controlled environment experiments.
**Table S4:** Population scores (loadings) from the climate and trait PCA.
**Table S5:** SNPs from the AMM GWAS. Significant SNPs are highlighted in orange.
**Table S6:** Trait and climatic parameters of MYB59 haplotype groups.

## Data Availability

The data that supports the findings of this study are available in the Supporting Information of this article.
