# Supplementary material for: MYB59 is Linked to Natural Variation of Water‐Use Associated With Warmer Temperatures in Arabidopsis thaliana
Source: Plant Environ Interact. 2026 Aug 9;7(4):e70199. doi: 10.1002/pei3.70199 (PMC13454550; doi:10.1002/pei3.70199)
Supplement: Supplementary file 2 [file PEI3-7-e70199-s002.pdf]

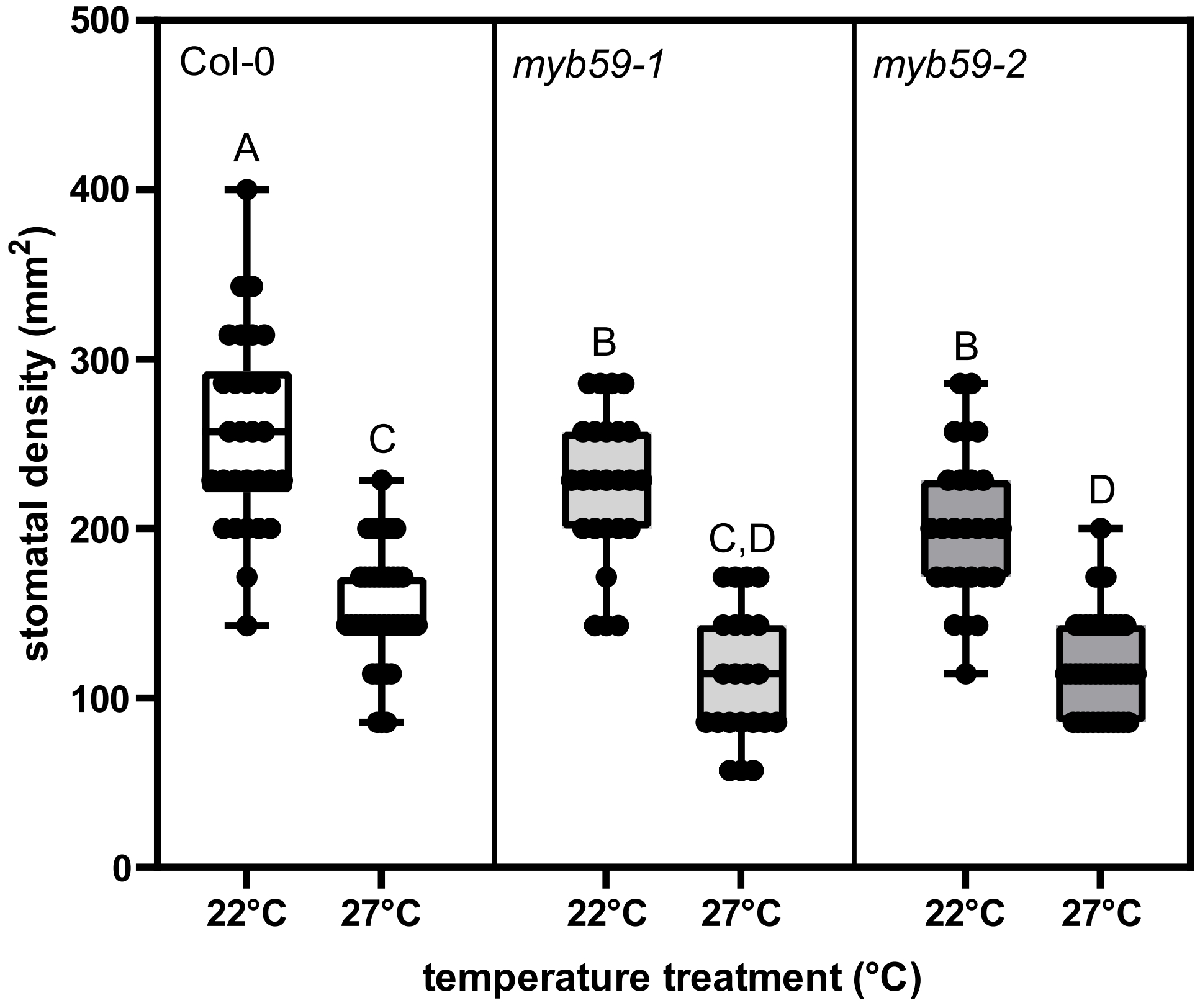

Figure S8 Ferguson et al
